# Adbuctor digiti minimi

**Published:** 2018-07-27

**Authors:** Daniel Turski

**Affiliations:** 1 Michael G. DeGroote School of Medicine, McMaster University, Ontario, Canada

**Figure:**
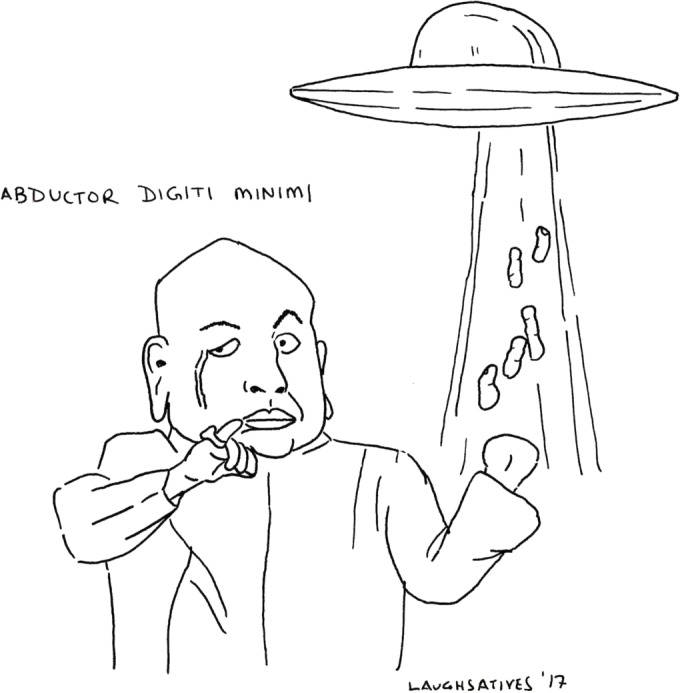


Medicine largely relies upon flexible, nimble, and agile thinking while working through complex differentials. Consequently, I like letting my mind roam while learning so as to enhance my mental flexibility. And this was one of the many images that popped into my mind while learning the musculoskeletal system. I’m also a visual learner.

*Daniel Turski is a second year medical student at McMaster University whose educational background is in medieval philosophy and linguistics. In addition to medical studies, Daniel is involved with national research projects investigating medical learner mental health, the Canadian Federation of Medical Students and the Ontario Medical Students’ Association as McMaster’s Senior Vice President of External Affairs, local campus student initiatives, and medical art*.

